# A Study on the Oxidation Performance of Soil Chromium with Acid Birnessite and Cryptomelane

**DOI:** 10.3390/toxics13040262

**Published:** 2025-03-31

**Authors:** Wei Zhang, Huan Yang, Shaohong You, Xia Zhong, Pingping Jiang, Xudong Lan, Rui Ma

**Affiliations:** 1 Guangxi Key Laboratory of Green Preparation and Application of Inorganic Materials, Guangxi Science & Technology Normal University, Laibin 546199, China; youshaohong@glut.edu.cn (S.Y.); 18878281049@163.com (X.L.); 2 College of Environmental Science and Engineering, Guilin University of Technology, Guilin 541004, China; w_zhang0701@163.com (W.Z.); 18875122954@163.com (H.Y.); dajavuu@163.com (X.Z.); 3 College of Earth Sciences, Guilin University of Technology, Guilin 541004, China

**Keywords:** acid birnessite, cryptomelane, chromium, oxidation, submergence

## Abstract

Current research focuses more on redox of toxic Cr(VI), with less attention to Cr(III) changes in flooded soil. First, the structure of acid birnessite and cryptomelane was characterized by X-ray diffraction (XRD), scanning electron microscopy (SEM), X-ray photoelectron spectroscopy (XPS) and other test methods. This study investigated farmland soil in Yuxi, Yunnan Province, under flooding stress induced by the addition of two distinct concentrations of manganese oxides. Throughout the experiment, key physicochemical properties of the soil—including pH, redox potential (Eh), Cr(VI) concentration, and chromium speciation—were systematically measured and analyzed. Structural characterization demonstrated distinct morphological and surface area properties. Specifically, acid birnessite, with petal-like stacked spheres, has a specific surface area of 103.76 m^2^/g, while cryptomelane, strip-shaped, has an area of 95.92 m^2^/g. The submergence experiment yielded the following phenomena: (1) During the 60-day flooding experiment, soil amended with 0.5% or 1% acid birnessite or cryptomelane exhibited an increase in Eh compared to the control group. (2) At the end of the 60-day submergence period, the Cr(VI) concentration in the soil treated with 1% acid birnessite increased by 2.4 times compared to the control group. In addition, after 60 days, Cr(VI) concentrations in the soil exceeded 5 mg/L in soils with manganese oxide added to them. This study evaluates how manganese oxides oxidize Cr(III), aiding in assessing their environmental risks and long-term impacts on metal transformation. The findings help predict chromium behavior in farm soils and guide remediation strategies.

## 1. Introduction

As urbanization and industrialization significantly accelerate, environmental issues rise to the forefront of national concerns [1]. Previous studies have revealed that heavy metal pollution worldwide is intensifying [2,3,4], with chromium contamination being a significant factor damaging the natural environment and posing health risks to organisms [5]. Hexavalent chromium (Cr(VI)) can enter the human body through oral ingestion, dermal contact, and inhalation, inducing multi-organ toxic effects. Specifically, it may cause renal impairment, dermatological lesions, and respiratory tract damage, and it notably exhibits neurotoxicity [6,7,8]. When environmental Cr(VI) concentration exceeds 0.05 mg/L, it may disrupt physiological homeostasis. If incorporated into the food chain, severe health consequences such as contact dermatitis, acute rhinitis, tympanic membrane perforation, and lung carcinoma may ensue [9]. The sources of Cr(VI) in soil encompass anthropogenic activities, geological origins, and oxidation of Cr(III) [10]. Primarily, soil chromium pollution stems from human industrial activities such as ore refining, steel and alloy production, metal plating processes, leather tanning, wood preservation treatments, and the discharge of chromium-containing wastes and effluents [5]. Geological Cr(VI) usually arises from the weathering of chromate minerals, though this scenario is infrequent due to the rarity of chromate minerals. Cr(III) enters the environment in two ways: (1) through direct anthropogenic emissions of Cr(VI) [11] and (2) as a product of Cr(VI) pollutant solidification/stabilization treatment [12]. In soil environments, Cr(III) is a relatively safe, less mobile micronutrient, whereas Cr(VI) is a highly toxic and mobile pollutant, often present in the form of chromate [13]. The oxidation of Cr(III) to Cr(VI) by oxidants like manganese oxides, hydrogen peroxide (H_2_O_2_), and photochemically generated radicals exacerbates the status of chromium pollution [14].

Manganese oxides, as a natural oxidant [15], are widely distributed in the Earth’s crust and possess high reactivity [16], a large specific surface area (ranging from 50 to 300 m^2^/g), and high oxidation-reduction potential (>1.23 V) characteristics, and they are an important component of soil oxides [17]. Manganese oxides play different roles in different regions of soil (aerobic, anaerobic, and surface zones). For example, in the aerobic zone, dissolved Cr(III) can be oxidized on the surface of manganese oxides, especially in acidic conditions (pH < 6.0) [14]. Weaver et al. [18] found that seven types of MnOx have significant oxidation capacity for Cr(III). The presence of Mn(III) and Mn(IV) in manganese oxides confers robust oxidizing capacity, facilitating the oxidation of diverse redox-active metal contaminants, including Cd, Ni, Cr, and Pb [19,20,21].

Mn is the third most abundant transition metal on Earth after Fe and Ti [22]. Currently, extensive research has been conducted on both synthetic and natural crystalline manganese oxide structures. However, direct isolation and investigation of manganese oxides from soil matrices remain challenging [23]. Given these difficulties, this study adopts synthetically prepared acid birnessite and cryptomelane, which have well-developed synthesis methodologies, to ensure structural reproducibility and experimental controllability [24]. Manganese oxides are generally divided into layered structures, tunnel structures, and low-valent manganese oxides [25]. The majority of Mn(IV) oxides found in nature exhibit layered and tunnel structures, characterized by their high oxidizing capacity and large specific surface area [26]. Low-valent manganese oxides are easily oxidized and reduced, so it is difficult for them to exist stably in nature [27]. Birnessite is ubiquitously present in soils and sediments, whereas cryptomelane primarily originates from authigenic precipitation. This mineral is most abundantly found in supergene oxidation zones of manganese deposits, manganese-bearing crusts, and lateritic weathering profiles [28,29].

Therefore, this study selects the layered structure of acid birnessite and the tunnel structure of cryptomelane, which are common in nature, as the research objects to explore their oxidation capacity of Cr(III) under submergent conditions. By studying how manganese oxides drive Cr(III) to form Cr(VI), we can gain a deeper understanding of the role of oxidants in chromium speciation transformation, which is of great significance for controlling and reducing high-toxicity and high-mobility chromium pollution.

## 2. Materials and Methods

### 2.1. Experimental Soil Propertie

Soil samples were collected from farmland in Yuxi City, Yunnan Province, and the geographical site was located at 24°09′00″ N and 102°24′35.9″ E. Samples were collected from the surface layer (0–10 cm), corresponding to the upper part of a *Mollic horizon*. The soil samples underwent natural air drying. Following this, the dried soil was crushed using a rubber mallet and sieved through a 10-mesh screen. During the screening process, tweezers were used to manually remove impurities from the soil, including stones, plant residues, and other debris. After these pretreatment steps, the resulting soil samples were sealed for subsequent analysis. The physicochemical properties and various forms of chromium in the processed soil were determined, such as exchangeable form of chromium (EF-Cr), reducible form of chromium (RF-Cr), oxidizable form of chromium (OF-Cr), and residual form of chromium (ResF-Cr). The results are presented in Table 1.

The experimental setup was configured as follows: Each treatment group was established with 500 g of soil contained in 1000 mL polypropylene beakers as cultivation vessels. A constant 3 cm water layer was maintained above the soil surface throughout the submergence treatment. Different amounts of added manganese oxides (acid birnessite and cryptomelane) served as individual experimental groups, with 0.1%, 0.5%, and 1% of the soil mass being added to the soil, respectively. These were thoroughly mixed, and then 500 mL of a 400 mg/L CrCl_3_ solution was added to each experimental group. The systems were allowed to stabilize for 60 days, with water losses being replenished every two days using the gravimetric method. Soil samples were collected at 7 days, 14 days, 30 days, and 60 days to measure soil Eh, soil pH, chromium speciation, and Cr(VI) concentration. A control group with only Cr added and a blank group without any additions were also included, with three replicates for each group. Thus, the experiment consisted of eight treatment groups: (i) 0.1%-Bir, (ii) 0.5%-Bir, (iii) 1%-Bir, (iv) 0.1%-Cry, (v) 0.5%-Cry, (vi) 1%-Cry, (vii) Cr (control with Cr only), and (viii) CK (blank control).

### 2.2. Synthesis of Acid Birnessite and Cryptomelane

#### 2.2.1. Synthesis of Acid Birnessite

Precisely 31.607 g of KMnO_4_ was dissolved in ultrapure water to prepare a 0.4 mol/L KMnO_4_ solution. Subsequently, a mixed solution of concentrated HCl and water was prepared in a 4:3 volume ratio. The KMnO_4_ solution was placed in a constant-temperature oil bath set at 130 °C, and a stirrer was introduced. After reaching boiling, the stirring speed was gradually increased to 300 revolutions per minute (rpm). A peristaltic pump was employed to slowly infuse the HCl solution at a rate of 0.7 mL/min. Upon completion of infusion, heating was continued for an additional 20 min. Following the heating period, the solution was allowed to cool naturally to room temperature. The solution was then transferred to a constant temperature oven set at 60 °C for aging for 12 h. The main drugs in the experiment are shown in Table 2.

#### 2.2.2. Synthesis of Cryptomelane

Precisely 11.062 g of KMnO_4_ was dissolved in ultrapure water to prepare a KMnO_4_ solution with a concentration of 0.4375 mol/L. Subsequently, 100 mL of a 1 mol/L MnSO_4_ solution was mixed with 100 mL of a 4 mol/L CH_3_COOH solution. Both solutions were placed in a constant-temperature oil bath and heated to 60 °C before being combined. Upon reaching the desired temperature, stirring was initiated at a speed of 300 (rpm). Heating was continued until the solution reached boiling point, and this boiling state was maintained for an additional 20 min. Following the completion of heating, the heat source was switched off, and the solution was allowed to cool naturally to room temperature. Subsequently, the solution was transferred to a constant-temperature oven set at 50 °C for aging for 24 h. After the aging process, the supernatant was discarded, and the precipitate was washed using a vacuum filtration system until the filtrate reached neutrality. Finally, the washed precipitate was dried and passed through a 200-mesh sieve for screening, and the resultant product was sealed for storage to prevent contamination. The main drugs in the experiment are shown in Table 2. All chemicals were of analytical grade.

### 2.3. Structural Characterization and Surface Property Analysis of Manganese Oxide

#### 2.3.1. X-Ray Diffraction (XRD)

In order to study the crystal structure of the sample, multi-function XRD (X ‘perT3 power, Eindhoven, The Netherlands) was used in this experiment, and the test conditions were as follows: Cukα radiation, tube flow of 40 mA, voltage of 45 kV, scanning angle range of 5–90°, scanning step size of 0.026°, and scanning speed of 10°/min. Phase identification was achieved by matching peaks to the International Centre for Diffraction Data (ICDD) PDF-4+ 2023 database.

#### 2.3.2. Scanning Electron Microscope (SEM)

In order to observe the morphological characteristics of the samples, SEM (SU 5000, Tokyo, Japan) was used in this experiment, and the acceleration voltage was 15 kV. Prior to observation, the samples were dried at 80 °C for 12 h and treated with gold spray to enhance electrical conductivity. Then, they were sputter-coated with a 5 nm layer using a Quorum Q150T ES coater (Quorum Technologies Ltd., Laughton, East Sussex, UK) to minimize charging effects. High-resolution images were captured at magnifications ranging from 5000× *g* to 50,000× *g*.

#### 2.3.3. Transmission Electron Microscopy (TEM)

In order to further reveal the crystal structure, morphology, and crystal surface spacing of manganese oxides, TEM (Talos™ F200X G2, Waltham, MA, USA) was used at an accelerated voltage of 200 kV. Samples were ultrasonically dispersed in anhydrous ethanol (99.9%) for 30 min (40 kHz, 100 W) and dripped onto copper wire to dry.

#### 2.3.4. X-Ray Photoelectron Spectroscopy (XPS)

In order to analyze the elemental morphology and content of each sample, the determination was performed using an XPS (Thermo Scientific K-Alpha, Waltham, MA, USA). The samples were placed on the sample tray and sent to the analysis chamber at a pressure of less than 2.0 × 10^−7^ mbar. The test conditions include spot size of 400 μm, operating voltage of 12 kV, filament current of 6 mA, full spectrum scanning capacity of 150 eV, and step size of 1 eV. The narrow-spectrum scan has a capacity of 50 eV and a step size of 0.1 eV to obtain detailed element information.

#### 2.3.5. Brunauer–Emmett–Teller (BET)

In order to analyze the specific surface area and pore structure of the samples, an automatic specific surface area and porosity analyzer (Micromeritics APSP 2460, Norcross, GA, USA) was used for each sample. Before the test, the samples were pretreated at 200 °C under vacuum for 6 h to remove surface adsorbents. Subsequently, N_2_ adsorption/desorption test was carried out in 77 K liquid nitrogen environment, and the total specific surface area of the material was calculated by BET method, and the integrated mesoporous analysis was realized.

#### 2.3.6. Fourier Transform Infrared Spectroscopy Analysis (FTIR)

In order to analyze the functional group composition of the samples, a FTIR (Thermo Scientific Nicolet iS20, Waltham, MA, USA) was used for testing. Prior to testing, 1 mg of each sample was mixed with 200 mg KBr powder and fully ground in an agate mortar in an infrared baking chamber. After the mixture was laminated into transparent sheets, it was scanned using a spectrometer with a wavelength range of 400–4000 cm^−1^ to obtain infrared spectral data of the sample.

### 2.4. Analysis of Physical and Chemical Properties of Soil

Soil pH was measured potentiometrically using a calibrated pH meter (PHS-3E, INESA Scientific Instrument Co., Ltd., Shanghai, China). Soil Eh was measured in situ using a portable oxidation-reduction potential (ORP) meter (HI98121, Hanna Instruments, Shanghai, China). Soil organic matter content was determined via the potassium dichromate oxidation/external heating method. Soil CEC was determined following the Chinese national standard HJ 889-2017: Soil Quality—Determination of Cation Exchange Capacity—Co (NH_3_)_6_]Cl_3_ Extraction/Spectrophotometric Method [30]. Soil Cr(VI) content was determined following the Chinese national standard HJ 1082-2019: Soil and Sediment—Determination of Hexavalent Chromium—Alkaline Digestion/Flame Atomic Absorption Spectrophotometry [31]. The soil chromium speciation extraction was performed using a modified BCR sequential extraction method [32].

### 2.5. Quality Control of the Analytical Data

All synthesis experiments were independently repeated 3 times. In order to ensure the accuracy of the test results, the instruments were cleaned and debugged before the test. During the analysis, standard soil samples and blank samples were added for quality control (one quality control sample was added for every 10 samples), and the sample recovery rates were between 85% and 110%. All water used was ultra-pure water. The quality control results meet the national standard, and the deviation is controlled within 10%.

### 2.6. Data Statistics and Analysis

The data in this study were preprocessed using Microsoft Excel 2019, and statistical analyses used SPSS 27. For significance testing of differences, the Least Significant Difference (LSD) method was adopted. Additionally, the creation of relevant data graphs and charts was facilitated by Origin 2021.

## 3. Results

### 3.1. Characterization of Acid Birnessite and Cryptomelane

#### 3.1.1. XRD Features

**Figure 1:**
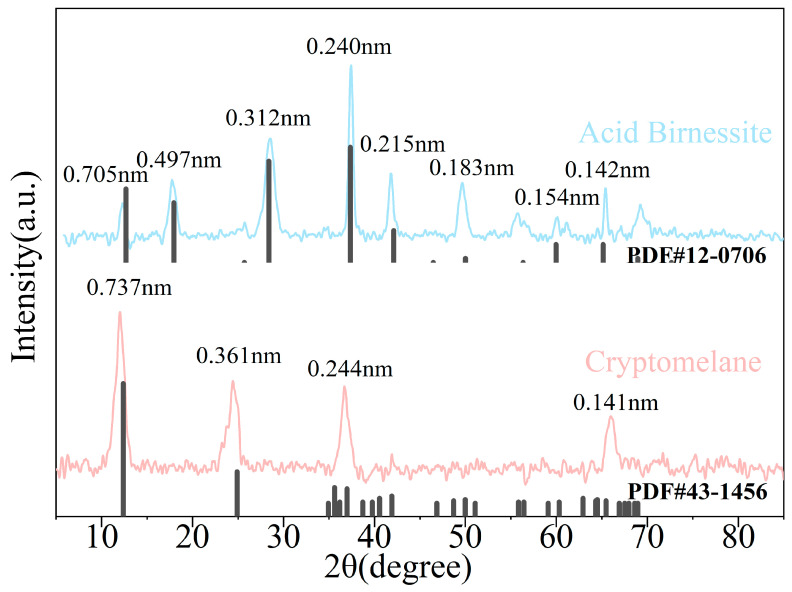
XRD pattern of acid birnessite and cryptomelane.

**Figure 2:**
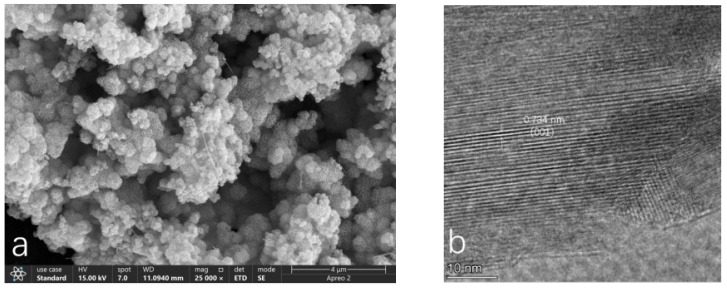
SEM/TEM images of the acid birnessite synthesized in the laboratory: (**a**) SEM images; (**b**) TEM images.

XRD was used for qualitative analysis of the acid birnessite. As shown by Figure 1, characteristic diffraction peaks were observed at angles of 5–80° with d values of 0.737 nm, 0.361 nm, 0.244 nm, and 0.141 nm, corresponding to crystal planes of (001), (002), (11-1), and (020), which are consistent with the characteristic diffraction peaks of acid birnessite and agree with PDF#43-1456. The synthesized material was determined to be layered acid birnessite by SEM and TEM images of the acid birnessite (Figure 2) [21].

As shown by Figure 1, characteristic diffraction peaks were observed at angles of 10–80° with d values of 0.705 nm, 0.497 nm, 0.312 nm, 0.240 nm, 0.215 nm, 0.183 nm, 0.154 nm, and 0.142 nm, corresponding to crystal planes of (110), (200), (310), (211), (301), (411), (521), and (002), which are basically consistent with the characteristic diffraction peaks of cryptomelane (PDF#12-0706), indicating that the synthesized product is cryptomelane.

#### 3.1.2. SEM/TEM Images

In the SEM morphological image magnified 25,000 times (Figure 2a), the morphological characteristics of acid birnessite can be clearly observed. It can be seen that the synthesized acid birnessite is composed of hydrangea-like aggregates with a size of approximately 0.5 μm, featuring a surface with multiple folds and irregular pores, and presenting as a single mineral phase. The TEM morphology image (Figure 2b) provides a very clear view of the lattice patterns of the acid birnessite. By processing the morphology image, the labeled crystal plane is determined to be 0.734 nm (001).

**Figure 3:**
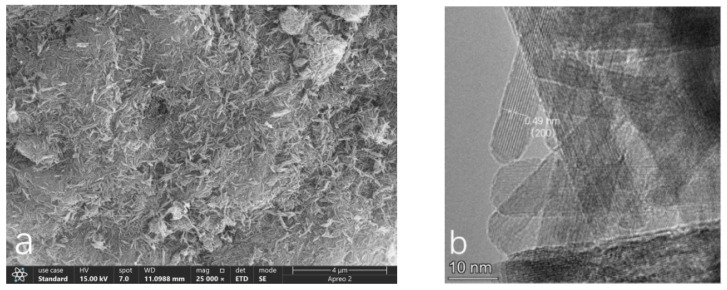
SEM/TEM images of the cryptomelane synthesized in the laboratory: (**a**) SEM images; (**b**) TEM images.

The SEM micrograph (Figure 3a), magnified 25,000 times, reveals the distinct morphological features of cryptomelane. Within the image, strip-like crystals of varying sizes are visible, with lengths of approximately 150 nanometers and widths of around 5 nanometers. Figure 3b offers a clear view of the lattice stripes of the cryptomelane. Upon processing the morphology image (Figure 3b), it is determined that the lattice spacing is 0.49 nanometers, indicating that the labeled crystal plane is (200).

#### 3.1.3. BET

**Figure 4:**
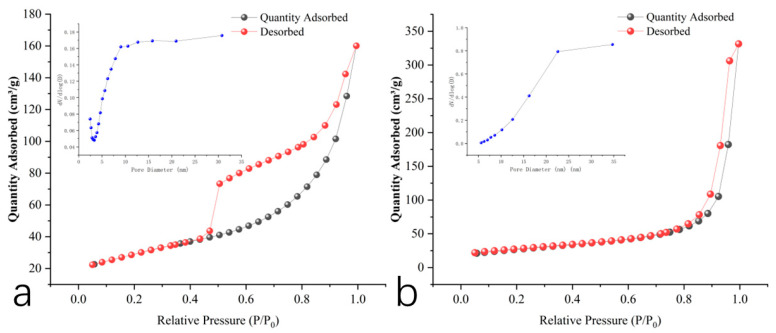
N_2_ adsorption/desorption isotherms of acid birnessite and cryptomelane, with pore size distribution plots located in top left corner: (**a**) acid birnessite; (**b**) cryptomelane.

Integrated mesoporous analysis of two materials was conducted using the BET methodology. The N_2_ adsorption/desorption isotherm and pore size distribution plot of acid birnessite reveal that the sample exhibits well-closed adsorption/desorption curves, belonging to Type IV isotherms according to the IUPAC classification, with an H4-type hysteresis loop. The BET test yields the following data: a BET specific surface area of 103.76 m^2^/g, a total pore volume of 0.247 cm^3^/g determined by the single-point method, and an average pore diameter of 9.54 nm. The shape of the adsorption/desorption curves indicates the presence of irregular pore structures in the sample (Figure 4), which aligns with the findings observed in the SEM morphology images. The BET results demonstrate that the synthesized acid birnessite possesses a large specific surface area and strong adsorption capacity.

The N_2_ adsorption/desorption curves and pore size distribution plot of cryptomelane are shown in Figure 4b. The sample’s adsorption/desorption curves are well closed and belong to Type IV isotherms, with an H3-type hysteresis loop according to the IUPAC classification, without showing adsorption saturation in the higher relative pressure region. The BET analysis provides the following data: a BET specific surface area of 95.92 m^2^/g, a total pore volume of 0.512 cm^3^/g determined by the single-point method, and an average pore diameter of 21.35 nm.

#### 3.1.4. XPS

**Figure 5:**
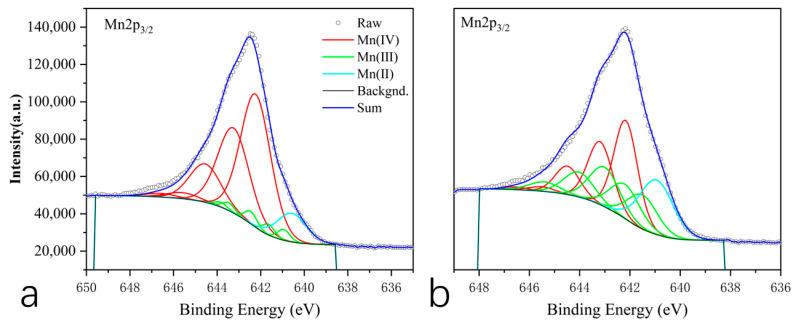
XPS Mn2p_3/2_ peak-fitting diagrams for acid birnessite and cryptomelane: (**a**) acid birnessite; (**b**) cryptomelane.

The Mn species in acid birnessite were determined using XPS. Charge correction was performed using the C1s peak with the assistance of Avantage 5.9931. The binding energy of Mn2p_3/2_ was observed at 642.50 eV, exhibiting a split peak that was asymmetric, which is characteristic of the standard Mn(IV) peak shape, and no Mn metal was detected. Previous reports have indicated the presence of both Mn(III) and Mn(IV) in both materials; therefore, fitting was conducted by incorporating multiple split peaks (Figure 5), with peak data sourced from Biesinger et al. [33].

In acid birnessite, the proportions of Mn(IV), Mn(III), and Mn(II) within the material were 85.24%, 6.03%, and 8.73%, respectively. For cryptomelane, the proportions of Mn(IV), Mn(III), and Mn(II) were 43.74%, 39.45%, and 16.81%, respectively.

#### 3.1.5. FTIR Spectroscopy

**Figure 6:**
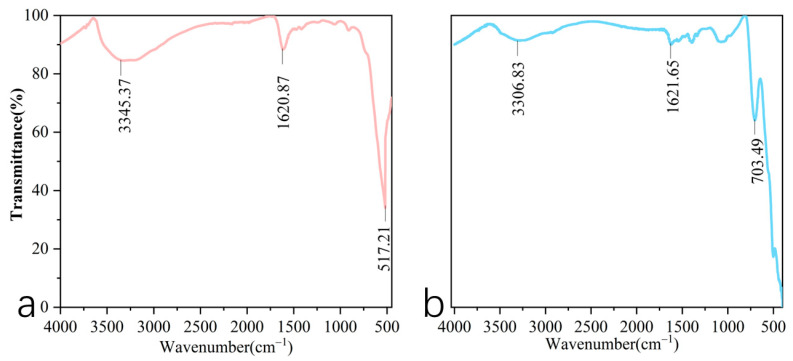
Fourier transform infrared (FTIR) spectra of acid birnessite (**a**) and cryptomelane (**b**): (**a**) acid birnessite; (**b**) cryptomelane.

According to the Fourier transform infrared (FTIR) spectra of acid birnessite (Figure 6a), the absorption vibration peaks of the sample are located at 3345.17 cm⁻^1^, 1620.87 cm⁻^1^, and 517.21 cm^−1^.

For cryptomelane, the absorption vibration bands are observed around 3306.83 cm^−1^, 1621.65 cm^−1^, and 703.49 cm^−1^ (Figure 6b).

### 3.2. Submergence Experiment

#### 3.2.1. Effect of Submerged Soil on pH and Eh Changes

**Figure 7:**
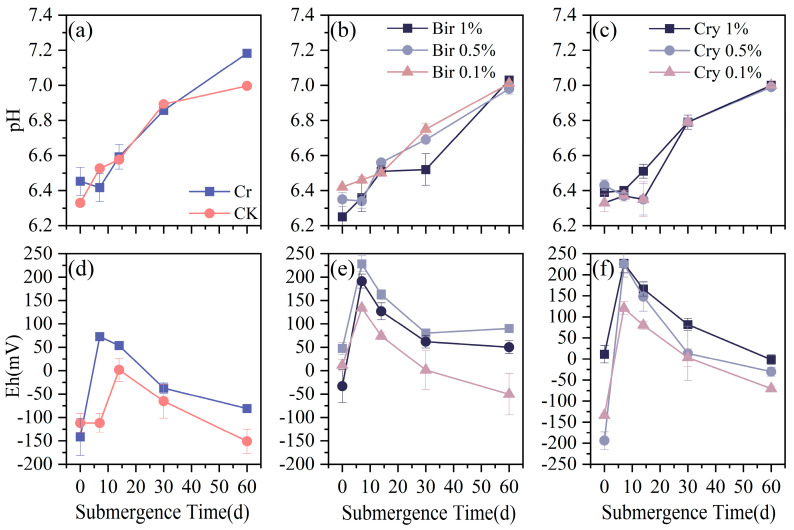
Changes in soil pH and Eh over time during submergence with different materials and amounts added: (**a**,**d**) Cr and CK (control); (**b**,**e**) acid birnessite; (**c**,**f**) cryptomelane.

In the 60-day soil submergence experiment, the effects of different types and amounts of manganese oxide on soil pH and Eh exhibited clear trends from Figure 7. Over time, the soil pH gradually stabilized and ultimately tended towards neutrality. Meanwhile, the Eh values of the soils, excluding the control group, showed an initial increasing trend during the early stages of submergence, peaking on the 7th day.

This phenomenon is influenced by multiple factors, resulting in a temporary increase in Eh values. After the 7th day, the Eh values of the submerged soils began to gradually decrease, reflecting the ongoing decomposition of organics and oxidation processes, which led to a decrease in oxidizable substances in the soil, and consequently, a reduction in Eh values.

#### 3.2.2. Effect of Submergence Condition on Cr(VI) Concentrations During the Submergence Phase

**Figure 8:**
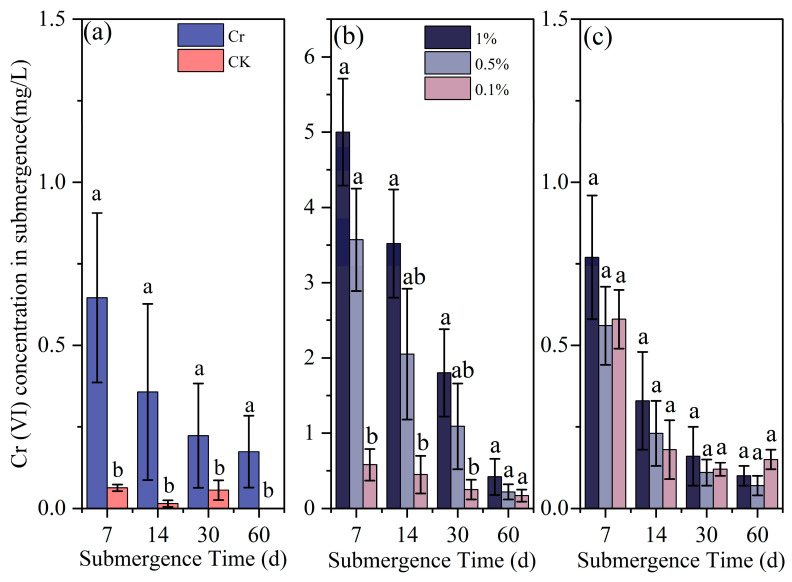
Effect of Cr (VI) concentration in submergence with different materials and additives over time: (**a**) Cr and CK (control); (**b**) acid birnessite; (**c**) cryptomelane. Different letters indicate significant differences between treatment groups (*p* < 0.05).

As shown in Figure 8, the submergence Cr(VI) concentrations in the eight treatment groups generally exhibited a gradual decreasing trend, ultimately falling below 0.5 mg/L. Specifically, compared to the blank group, the addition of Cr(III) significantly increased the submergence Cr(VI) concentration in the control group (*p* < 0.05). At 7d, 14d, and 30d, 1%-Bir significantly elevated the submergence Cr(VI) concentration compared to 0.1%-Bir (*p* < 0.05). At 7d, the submergence Cr(VI) concentration in the 1%-Bir group reached 5 mg/L, representing a 7.73-fold increase compared to the control group. There was no significant correlation between the submergence Cr(VI) concentration and different amounts of added cryptomelane (*p* > 0.05).

#### 3.2.3. Effect of Submergence Condition on Cr(VI) Concentration in Soil

**Figure 9:**
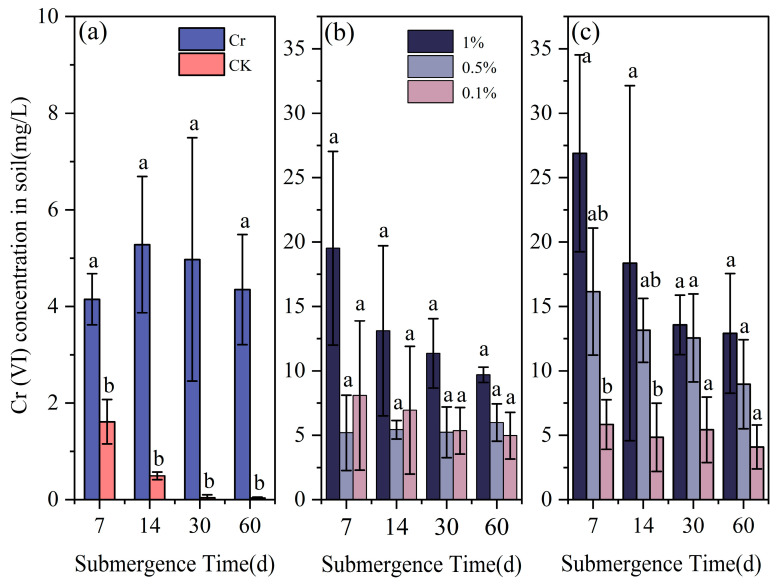
Effect of Cr (VI) concentration in soil with different materials and additives over time: (**a**) Cr and CK (control); (**b**) acid birnessite; (**c**) cryptomelane. Different letters indicate significant differences between treatment groups (*p* < 0.05).

As shown in Figure 9, the soil Cr(VI) concentration in the system roughly inversely correlates with time.

The addition of Cr(III) has a significant impact on soil Cr(VI) concentration (*p* < 0.05), with the Cr(VI) concentration in the control group reaching 5.28 mg/L on day 14. In the 1%-Bir group, the Cr(VI) concentration peaked at 19.52 mg/L on day 7, while in the 1%-Cry group, it reached 26.89 mg/L on the same day. Within the system where cryptomelane was added, 1%-Cry significantly increased the Cr(VI) concentration compared to 0.1%-Cry during the 0–14-day period (*p* < 0.05). However, there was no significant correlation between the amount of cryptomelane added and Cr(VI) concentration at 30 and 60 days (*p* > 0.05). In the system with acid birnessite added, there was no significant correlation between soil Cr(VI) concentration and the amount added (*p* > 0.05). From the figure, it can be observed that the soil Cr(VI) concentration in the Cry system is slightly higher than that in the Bir system, which is related to the differences in their crystal structures.

#### 3.2.4. Effect of Submerged Soil on the Forms of Soil-Bound Cr

Focusing solely on the total concentration of heavy metals overlooks crucial factors such as their different forms and mobility in soil, which significantly influence their environmental behavior and toxicity. Hence, the formal changes in chromium were analyzed using BCR morphology sequential extraction in a 60-day submergence experiment [34].

**Figure 10:**
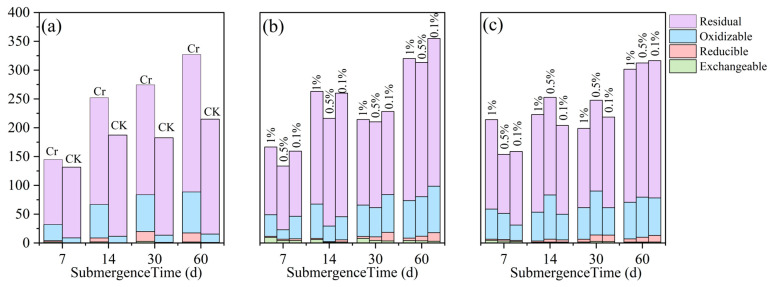
Effect of soil chromium formations with different materials and additions over time: (**a**) Cr and CK (control); (**b**) acid birnessite; (**c**) cryptomelane.

The measurement results of chromium species are shown in Figure 10, indicating that chromium primarily exists in the residual form in soil. At 60 days, the residual form accounted for approximately 75% of the total chromium concentration. In both the Cr(III) and CK systems, the concentrations of total chromium, reducible chromium, oxidizable chromium, and residual chromium positively correlated with the submergence duration. In the Bir and Cry systems, the concentrations of these chromium species exhibited an upward trend during the first 7 and 14 days of submergence. Between 14 and 30 days, the chromium concentrations of all species fluctuated slightly, while the residual chromium concentration peaked at over 200 mg/kg at 60 days. During the mid-phase of the experiment (14–30 days), the total chromium content in the systems with material additions was lower than that in the control group, primarily reflected in the reducible form, and the reducible chromium content inversely correlated with the amount of material added. After 60 days of submergence, the total chromium concentrations in all eight treatment groups converged to approximately 300 mg/kg, with the oxidizable form accounting for about 20% of the total chromium concentration, and the reducible and exchangeable forms contributing less than 5% of the total chromium concentration. In soils, the oxidizable form of Cr is initially facilitated by manganese oxides, while its dynamics during the mid-to-late stages (14–60 days) are primarily governed by the concentration of supplemented Cr(III).

## 4. Discussion

Based on the SEM and TEM images in Figure 2, the synthesized compound is identified as layered acid birnessite with a lamellar structure. Figure 2a reveals the presence of some rod-shaped crystals, which are likely to be minor precursors (hexagonal manganite) or tunnel-structured acid birnessite produced during the synthesis process [35,36]. The fitting results of the XPS spectrum of acid birnessite (Figure 5a) indicate that the proportions of Mn(IV), Mn(III), and Mn(II) are 85.24%, 6.03%, and 8.73%, respectively. Limited Mn^3+^/Mn^2+^ content (6.03% and 8.73%, respectively) minimizes self-reduction reactions (e.g., Mn^4+^ → Mn^3+^), preserving the oxidative capacity and sustaining high Eh levels even under waterlogged conditions. Evidently, the Mn in the synthetic acid birnessite is fully oxidized, with Mn(IV) exhibiting the strongest oxidizing capacity. The absorption bands at 3345.17 cm^−1^ and 1620.87 cm^−1^ are commonly associated with the stretching and bending vibrations of OH groups, respectively [37]. For inorganic compounds, these vibrations may arise from adsorbed or crystalline water in the sample. The absorption band at 517.21 cm^−1^ is typically related to metal–oxygen (M-O) stretching vibrations and is a characteristic absorption band of layered acid birnessite [38]. This confirms that acid birnessite consists of a layered mineral formed by the alternating stacking of MnO_6_ octahedra and water molecules [37]. This structural flexibility enhances electron transfer during Cr^3+^ oxidation (Cr^3+^ → Cr^6+^), as Mn^3+^/Mn^4+^ edge sites dynamically adjust bond lengths.

The SEM and TEM images in Fig. 3 identify the synthesized compound as tunnel-structured cryptomelane. The adsorption/desorption curve in Figure 4b suggests an irregular pore structure distribution on the surface of cryptomelane. BET calculations reveal a large specific surface area, average pore diameter, and total pore volume. As observed in Figure 7 and Figure 8, during the initial flooding stage (0–7 days), acid birnessite, with a higher specific surface area, exhibited greater efficiency in oxidizing Cr(III) to Cr(VI) compared to cryptomelane. The fitting results of the XPS spectrum of cryptomelane (Figure 5b) show that Mn(IV), Mn(III), and Mn(II) account for 43.74%, 39.45%, and 16.81% of the material, respectively. Higher Mn^3+^/Mn^2+^ content (39.45% and 16.81%) indicates a reduced oxidative potential. Mn^3+^ may act as an electron donor (Mn^3+^→ Mn^2+^) in redox reactions, partially counteracting Cr(III) oxidation and resulting in weaker Eh elevation. Notably, the Mn(III) content far exceeds that of the acid birnessite. While Mn(IV) has a stronger oxidizing capacity than Mn(III), Mn(III) can increase the oxidation rate of the material [39]. Therefore, the oxidation rate of cryptomelane should be higher than that of acid birnessite. The absorption bands at 3306.83 cm^−1^ and 1621.65 cm^−1^ are usually related to the stretching vibrations of O-H bonds, and the remaining absorption vibration band located around 703.49 cm^−1^ is a characteristic peak of Mn-O bond stretching vibrations in cryptomelane-type compounds, indicating the presence of abundant surface hydroxyl groups [40]. The stronger and more ordered Mn–O bonds reduce structural adaptability, slowing redox kinetics and limiting Cr^3+^ oxidation efficiency.

After submergence, significant changes occur in the soil environment. In submerged soil, the original oxygen is gradually consumed by microbial activity, resulting in a gradual decrease in soil Eh and concurrent reduction of soil iron oxides, which tends to neutralize the soil pH [41,42]. This phenomenon is unrelated to the types and amounts of manganese oxide added. This suggests that although manganese oxides may affect soil properties in other ways, they do not alter the trend of soil pH neutralization after long-term submergence. Since the redox potential of Cr(VI) is greater than that of Cr(III), in the initial stage, the concentration of Cr(VI) gradually increases due to the oxidation of Cr(III) by manganese oxides, accompanied by a gradual increase in Eh values. In the mid-to-late stages, microbial activity dominates, accelerating the decomposition of organics and lowering soil Eh [41]. Meanwhile, under low redox potential conditions, Cr(VI) is easily reduced to Cr(III), which is evident in the subsequent two experiments. Marschner also indicated that in submergent soil, the original oxygen is consumed by microbial respiration, leading to a transition from an oxidized to a reduced state. Therefore, the Eh value of the soil in a submergent anaerobic environment ultimately decreases [41]. The Eh values of all systems generally show an initial increase followed by a decrease. Among them, systems with added manganese oxides have higher Eh values compared to the control group, indicating that the addition of manganese oxides increases soil Eh values. The reason for the Eh increase in the control group only after 7 days may be that the originally present soluble oxidizing substances in the soil do not dissolve, precipitate, and diffuse within a short time. After a brief stable period, the dissolution and migration of soluble oxidizing substances lead to an increase in Eh.

In the early stage of the experiment (0–14 days), the soil was acidic, and both acid birnessite and cryptomelane surfaces carried negative charges, resulting in fast oxidation rates and significantly higher Cr(VI) concentrations in submergent conditions and soil compared to the control group. In the 0.1%-Cry group, the Cr(VI) concentration in submergent soil increased by 0.03 mg/L at day 60, which is similar to the results of Lyu et al. [43] and Ao et al. [44] and also shows a slow increase in Cr(VI) concentration in the later stages of submergence. As Eh decreases and the soil tends towards a reduced state, the morphology of iron oxides changes from amorphous with strong adsorption capacity to crystalline with relatively weak adsorption capacity. Among them, amorphous iron oxides have a larger specific surface area and can more effectively bind with heavy metal ions to form coprecipitates, which affects the leaching concentration of chromium in soil, usually resulting in a decrease in leaching concentration [45,46]. Under submergence conditions, Cr(III) in soil with added acid birnessite can continuously dissolve and be oxidized. Moreover, adding 1% acid birnessite significantly increases the Cr(VI) concentration in submerged soil compared to adding 0.1% acid birnessite. After 30 days, the experimental system is in a reduced state, which can continuously reduce the dissolved Cr(VI) to Cr(III), ultimately leading to a continuous decrease in Cr(VI) concentration in soil [41]. Studies have shown that hydroxyl radicals (•OH) produced by the photolysis of Fe(OH)^2+^ in surface soil can further oxidize Cr(III) to Cr(VI) [17]. In addition, during experimental determination of Cr(VI) in submerged soils, the layered structure of acidic birnessite may enhance exposure of Mn^3+^/Mn^4+^ edge sites, which serve as active centers for redox reactions [26,28]. This structural feature improves contact efficiency with Cr^3+^ ions by increasing the accessible surface area and catalytic sites. In soil environments, controlled cation intercalation into the interlayer structure is crucial for acidic birnessite’s phase transformation, which may result in the transformation to hausmannite or manganite [35,36]. This leads to a decrease in its oxidation properties. The presence of Cr(III) induces crystal distortion and agglomeration in cryptomelane. Following Cr(III) oxidation by cryptomelane, the material stability is enhanced.

In soil, chromium exists in four forms: exchangeable, reducible, oxidizable, and residual. Among them, the exchangeable form is considered the most bioavailable and biologically active [47]. The reducible and oxidizable forms are less active but can convert into the more mobile exchangeable form under acidic conditions. The residual form is inactive in soil, usually fixed in the soil and not easily bioavailable [48]. The residual form of Cr (60–70%) dominates, suggesting lower immediate toxicity but potential slow release under future oxidative perturbations (e.g., soil drying or O_2_ influx). Soil chromium speciation experiments show that in the early stage of the experiment (0–7 days), the total chromium concentration in systems with added manganese oxides is higher than that in the Cr system, primarily reflected in the oxidizable form. Firstly, the dissolution of Cr(OH)_3_ is inhibited when pH > 6 [49,50]. Secondly, manganese oxides can adsorb Cr(III). As the experiment progresses, the exchangeable form gradually converts to the residual form, resulting in a gradual decrease in both submergence and soil Cr(VI) concentrations. Elevated Cr(VI) in submerged acid birnessite soils suggests higher chromium mobility and toxicity risks, as Cr(VI) is water-soluble and readily bioavailable. In contrast, cryptomelane’s limited Cr(VI) generation implies lower environmental hazards under fluctuating moisture conditions. Manganese oxides can reduce or inhibit the production of reducible Cr in soil throughout the experiment (0–60 days), but their inhibitory effect weakens over time.

## 5. Conclusions

This paper characterizes manganese oxides through methods such as XRD, SEM, XPS, and BET; explores the changes in chromium in soil under submergence conditions; and predicts the speciation, toxicity, and chemical behavior of chromium during their biogeochemical cycling in the environment. Manganese oxides, serving as efficient oxidants and adsorbents, are studied in this paper through acid birnessite and cryptomelane to investigate the interaction between manganese oxides and redox-active chromium and the transformation of soil Cr under submergence conditions, providing a theoretical basis for chromium-contaminated sites. The results indicate the following:

(1) In the material characterization experiments, the two materials were qualitatively analyzed using X-ray diffraction (XRD) and compared with standard cards to confirm the successful synthesis of the target materials, namely acid birnessite and cryptomelane. Morphological observations through SEM and TEM revealed that acid birnessite consists of lamellar crystals stacked into hydrangea-like shapes, while cryptomelane crystals are strip-shaped. XPS analysis of the Mn species in the samples showed that both acid birnessite and cryptomelane are Mn(IV) oxides. The specific surface areas of acid birnessite and cryptomelane are 103.76 m²/g and 95.92 m²/g, respectively.
(2) In the submergence experiments, soil pH positively correlated with submergence duration, while soil Eh negatively correlated with submergence duration. The soil submergence experiments demonstrated that altering soil properties and adding manganese oxides can affect Cr stability. Under acidic conditions, especially in soil environments containing acid birnessite, the submergence Cr(VI) and soil hexavalent chromium concentrations are affected. Under submergence conditions, compared to the control group with added Cr(III), 1% acid birnessite increased the submergence Cr(VI) concentration by 2.4 times and the soil Cr(VI) concentration by 2.23 times. Soil amended with 1% cryptomelane exhibited a 2.9-fold increase in Cr(VI) concentration compared to the Cr-only control group on 60 Day. The reason for this is that cryptomelane is relatively stable in the soil. After 60 days, the soil Cr(VI) concentration remained at >5 mg/L, exceeding the safety thresholds stipulated by some ecological and human health standards. Therefore, soil containing acid birnessite components results in higher submergence Cr(VI) concentrations under submergence conditions, while soil containing cryptomelane components leads to higher soil Cr(VI) concentrations. Both materials pose potential hazards to the ecological environment.
(3) Due to its strong oxidizing capacity and good adsorption properties for heavy metals, acid birnessite necessitates caution, as this mineral can oxidize the existing Cr(III) in soil to the more toxic Cr(VI). The formation of Cr(VI) increases the mobility and toxicity of chromium in the environment.

This study enriches the change trend of hexavalent chromium and the change in the physical and chemical properties of soil under flooding. By incorporating two ubiquitous soil minerals (acid birnessite and cryptomelane) with distinct redox-active structures, our experimental design enhances the practical relevance of the findings, as these minerals are widely distributed in natural and contaminated soils. Caution is advised in practical applications, and combining other remediation technologies or conducting environmental monitoring is recommended to ensure environmental safety. We look forward to future studies that will add to the use of plant uptake tests to assess the bioavailability of chromium under realistic soil moisture regimes.

## Figures and Tables

**Table 1:** Physical and chemical properties of tested soil.

pH	OM (%)	CEC (cmol/kg)	Available N (mg/kg)	Olsen-P (mg/kg)	EF-Cr (mg/kg)	RF-Cr (mg/kg)	OF-Cr (mg/kg)	ResF-Cr (mg/kg)
5.63	1.74	7.48	32.03	21.25	0.28	0.18	6.72	261.63

**Table 2:** The main drugs in the experiment.

Chemical Name	Purity/Concentration	Manufacturer
KMnO_4_	≥99.5%	Nanjing Chemical Reagent Co., Ltd. (Nanjing, China)
HCl	36–38%	Xilong Scientific Co., Ltd. (Shantou, China)
MnSO_4_·H_2_O	≥99.0%	Aladdin Biochemical Technology Co., Ltd. (Shanghai, China)
CH_3_COOH	≥99.7%	Shanghai Macklin Biochemical Co., Ltd. (Shanghai, China)

## Data Availability

The raw data supporting the conclusions of this article will be made available by the authors upon request.
